# Jiawei Runjing Decoction Improves Spermatogenesis of Cryptozoospermia With Varicocele by Regulating the Testicular Microenvironment: Two-Center Prospective Cohort Study

**DOI:** 10.3389/fphar.2022.945949

**Published:** 2022-08-09

**Authors:** Huang Liu, Zhongwang Huang, Houbin Zheng, Zhiyong Zhu, Hui Yang, Xingzhang Liu, Tao Pang, Liping He, Hai Lin, Lei Hu, Qingqi Zeng, Lanying Han

**Affiliations:** ^1^ The First School of Clinical Medicine, Nanjing University of Chinese Medicine, Nanjing, China; ^2^ NHC Key Laboratory of Male Reproduction and Genetics, Department of Andrology, Guangdong Provincial Reproductive Science Institute (Guangdong Provincial Fertility Hospital), Human Sperm Bank of Guangdong Province, Guangzhou, China; ^3^ Department of Andrology, Shenzhen Traditional Chinese Medicine Hospital, Shenzhen, China; ^4^ NHC Key Laboratory of Male Reproduction and Genetics, Department of Ultrasonography, Guangdong Provincial Reproductive Science Institute (Guangdong Provincial Fertility Hospital), Human Sperm Bank of Guangdong Province, Guangzhou, China; ^5^ NHC Key Laboratory of Male Reproduction and Genetics, Department of Clinical Laboratory, Guangdong Provincial Reproductive Science Institute (Guangdong Provincial Fertility Hospital), Human Sperm Bank of Guangdong Province, Guangzhou, China; ^6^ Department of Integrated Chinese and Western Medicine, Jiangsu Health Vocational College, Nanjing, China; ^7^ NHC Key Laboratory of Male Reproduction and Genetics, Department of Traditional Chinese Medicine, Guangdong Provincial Reproductive Science Institute (Guangdong Provincial Fertility Hospital), Human Sperm Bank of Guangdong Province, Guangzhou, China

**Keywords:** cryptozoospermia, varicocele, JWRJD, testicular microenvironment, network pharmacology

## Abstract

**Objective:** The aim of the study was to explore the evidence of JWRJD in the treatment of cryptozoospermia.

**Methods:** A total of 162 cryptozoospermia patients with varicocele who refused to undergo surgery were included from January 2021 to December 2021. They were divided into the Jiawei Runjing Decoction group (group A), tamoxifen group (group B), and no treatment group (group C), and after the follow-up for 3 months, therapeutic effectiveness was compared. Network pharmacology was used to analyze and validate the effects and mechanisms of JWRJD.

**Results:** Fifty-eight patients were treated with JWRJD, 55 with tamoxifen, and 49 without any treatment. After treatment, five patients were lost: one in group A, one in group B, and three in group C. The sperm count and the decrease of FSH in group A were significantly higher, but the degree of decline in the testicular volume and the degree of vein expansion have decreased significantly, which were closely related to the testicular volume (TV) [especially changes in the left testicular volume (ΔL-TV)], citric acid (CC) and its changes (ΔCC), and the vein width (VW) [especially left spermatic vein width (L-VW) and mean vein width (M-VW) and their changes (ΔL-VW and ΔM-VW)], as well as the sperm count before the treatment (bSC), which were the significant indexes to predict the therapeutic effect, especially for patients >35 years old and with grade III varicoceles. Network pharmacological analysis verifies that it can be regulated by fluid shear stress and the atherosclerosis pathway to improve the testicular microenvironment for spermatogenesis.

**Conclusion:** JWRJD may promote spermatogenesis in cryptozoospermia patients with varicocele, which may be closely related to improving the testicular microenvironment, especially for >35 year olds and grade III varicocele patients.

## 1 Introduction

The incidence of infertility in the world is approximately 8–12%, half of which is caused by males (Bay et al., 2013; Kazemijaliseh et al., 2015). Among the factors causing male infertility, abnormal sperms are the most common (Jafari et al., 2021). Cryptozoospermia is the extremely severe form of oligozoospermia, which is manifested as sperm that could not be found in routine microscopic examination of ejaculated semen but could be seen in the deposition after centrifugation (World Health Organization, 2010). Its etiology is complex and varied, and varicocele is one of the most common etiologies (Punab et al., 2017). Although surgery is the preferred treatment for varicocele (Caradonti, 2020), its expensive and invasive nature is less readily accepted by patients, especially those with cryptozoospermia, who are more willing to accept medication or forgo intervention and wait for assisted reproductive technology (French et al., 2008; Fine and Franco, 2015; Lima et al., 2020). In patients with low spermatogenesis with varicocele without surgical treatment, drugs are considered the first choice of treatment. Male infertility guidelines indicate that selective estrogen receptor modulators (SERMs) are considered an empiric treatment for male infertility, and the mechanism of action is to block the estrogen receptor activity so as to promote the luteinizing hormone (LH) and stimulate sperm production (Salonia et al., 2020). Tamoxifen is one of these most commonly used drugs, but its endocrine effects are controversial (Vandekerckhove et al., 2000). The search for more ideal oral drugs was the focus of improving patients with cryptozoospermia of varicocele.

Traditional Chinese medicine (TCM) has a history of more than 5,000 years in treating male infertility and childlessness (Zhou et al., 2019). TCM believes that infertility is the same as “Wu-zi” (*No child*), “Wu-hou” (*No offspring*), “Jing-xi” (*Sperm rarity*), and “Jing-leng” (*Sperm unfrequented*). The treatment of spermatogenesis with various Chinese herbs has different values from modern medicine (Wang et al., 2018; Zhou et al., 2019). In TCM, varicocele cryptozoospermia was considered to belong to the deficiency syndrome of “Qi-stagnation” *(Stagnation of the circulation of vital energy)*, “Blood-stasis” *(Coagulation of blood)*, and Shen-Yang (Kidney-Yang) (Hu et al., 2013). Good results could be obtained by activating *Qi* and *Blood*, removing stasis and clearing collaterals, and nourishing Shen-Yang (Kidney-Yang). The previous animal studies (Dong et al., 2021; Yang et al., 2021) had shown that Runjing Decoction (*RJD*) which includes 10 Chinese medicines [*Cuscuta chinensis* Lam*.* (CS), *Dioscorea polystachya* Turcz*.* (DR), *Polygonatum sibiricum* Redouté (PR), *Epimedium brevicornu* Maxim*.* (EF)*, Lycium barbarum* L. (LF), *Eleutherococcus senticosus* (Rupr. & Maxim.) Maxim. (AS), *Rhodiola crenulata* (Hook. f. & Thomson) H. Ohba (RC), *Cyathula officinalis* K.C.Kuan (CR), *Citrus × aurantium* L. (CRP), and *Hirudo* (Hd)] could promote blood circulation and clear collaterals, nourish *Shen-Yang* (*Kidney-Yang*) and other functions to improve the proliferation of mouse germ cells, and increase the number of sperms, but the clinical observation of RJD on male infertility still lacks effective evidence. For this reason, we improved the decoction on the basis of RJD and created the Jiawei Runjing Decoction (*JWRJD*) by adding two traditional Chinese medicines [*Homo Sapiens* (HS) and *Eupolyphaga seu Steleophaga* (ES)], which could be more powerful to strengthen the function of spermatogenesis and promote blood circulation and dredge collatulopathy. JWRJD was applied to patients with cryptozoospermia with varicoceles who were observed for 3 months. Finally, we found interesting results.

## 2 Materials and Methods

### 2.1 Patient Setting

A total of 162 patients with cryptozoospermia of varicocele were admitted to the male department of Guangdong Reproductive Hospital and Shenzhen Traditional Chinese Medicine Hospital from January 2021 to December 2021 due to male sterility. Here, 117 were from Guangdong Reproductive Hospital, and 45 were from Shenzhen Traditional Chinese Medicine Hospital. The age ranged from 25 to 54 years, with an average of 34.24 ± 5.51 years old; the body weight was 63.5–77.5 kg, with an average of 70.68 ± 2.99 kg. The height was 158–175 cm, with an average of 169 ± 3.23 cm; BMI was 21.71–27.94, with an average of 24.68 ± 1.22; primary infertility was seen in 123 people, and secondary infertility, in 39 people. This study has been registered through the Chinese Clinical Trial Registry (https://www.chictr.org.cn/) with the registration number (ChiCTR2200060463).

### 2.2 Inclusion Criteria

The inclusion criteria include the following: 1) meeting the WHO diagnostic criteria for sterility and cryptozoospermia (World Health Organization, 2010, 5th edition.). 2) B-ultrasound confirmed varicocele. 3) Consistent with the diagnosis of qi stagnation and blood stasis and *Shen-Yang* (*Kidney-Yang*) (Zhou et al., 2020). 4) Refused surgical treatment and acceptance of drug treatment and insisting on taking drug continuously for 30 days. 5) The spouse was normal and be able to ejaculate normally *in vitro* and collecting the semen. 6) Can cooperate with the examination, willing to accept the follow-up and sign informed consent.

### 2.3 Exclusion Criteria

The exclusion criteria include the following: 1) those who did not meet the inclusion criteria. 2) Chromosome abnormalities and azoospermia factor (AZF) gene deletion or other genetic abnormalities. 3) Close contact with toxic or radioactive substances in the last 6 months. 4) Had suffered from serious reproductive tract infection. 5) Tumor patients. 6) Patients with systemic immune diseases. 7) There was long-term drug use or alcohol addiction and other enthusiasts. 8) Sexual dysfunction or retrograde ejaculation.

### 2.4 Physical Check

All patients underwent a general physical examination which included a comprehensive examination of height, weight, vital signs, heart, liver, lungs, kidneys, bones, limbs, and nervous system at room temperature of 25°C. They also underwent the male specialist examinations which included secondary sexual characteristic development, external genitalia, testicular size, epididymis, vas deferens, and spermatic veins. The testicular size was measured with the Prader orchidometer with the values ranging from 1, 2, 3, 4, 5, 6, 8, 10, 12, 15, 20, and >25 ml (Krausz, 2011).

### 2.5 Treatment and Follow-Up

All patients were fully informed of treatment options, made free choices, and signed informed consent forms to select the treatment methods, including JWRJD or tamoxifen or gave up treatment. All of them were followed up for 3 months as a course of treatment. All the indicators in the semen and blood test were checked before and after treatment.

#### 2.5.1 JWRJD Group (Group A)

One dose every day, morning and evening, was taken continuously for 1 month, and semen and blood were checked before and after the treatment.

#### 2.5.2 Tamoxifen Group (Group B)

One tablet was taken in the morning and evening twice a day for 1 month, and then, semen and blood were checked (specification: 10 mg/tablet, Approval No.: GyzzH32021472, Yangzi River Pharmaceutical Group Co., Ltd.).

#### 2.5.3 Without Any Treatment Group (Group C)

Semen and blood were examined at the beginning and the end without any drug treatment.

### 2.6 Semen Detection

According to the semen detection standards in the fifth edition of the WHO Manual for Detection and Analysis of Human Semen (World Health Organization, 5th ed., 2010), fresh ejaculated semen within 2–7 days of abstinence was collected, the semen volume was measured by the weighing method, the pH value of the semen was measured by using a precision pH test paper, and the semen was liquefied in a 37°C water bath and then sent for microscopic examination. When no sperms were found, centrifugation was carried out at a speed of 3,000 g per minute for 15 min. The sediment was taken for microscopic examination, and the total number of sperm counts was calculated.

### 2.7 Seminal Plasma Biochemistry

The concentrations of neutral α-glucosidase, citric acid, and zinc in the seminal plasma were determined by using an automatic biochemical analyzer (*Mindray BS-350E*) and seminal plasma biochemical detection kit [seminal plasma α-glucosidase detection kit (kinetic method), R_2_: 1 × 10 ml; seminal plasma citrate detection kit (*End-point method*), specification 1: R_1_: 1 × 15 ml, R_2_ lyophilized powder: 1 × 4 ml, calibration product: 1 × 1 ml; and seminal plasma zinc detection kit (*PAN method*), R_1_: 1 × 20 ml. Xindi Biopharmaceutical, Nanjing].

### 2.8 Reproductive Hormones

Peripheral blood was collected at 9:00 a.m. and centrifuged at 1,000 rpm for 15 min. The upper serum was taken, and the concentrations of follicle stimulating hormone (FSH), luteinizing hormone (LH), prolactin (PRL), estradiol (E_2_), and testosterone (T) in the serum were determined by immunochemiluminescence (Roche, COBAS 8000, and COBAS e 602) and a kit (Elecsys FSH, LH, PRL, E_2_ and T, COBAS, Roche, Germany).

### 2.9 B-Ultrasound Examination

The patient was placed in the supine position at 25°C, and scrotal ultrasonography was performed with color ultrasound Doppler (Shenzhen Mindray Bio-Medical Electronics Co., Ltd; Resona8; the linear high-frequency 7–14 MHz probe was used), and the width of the spermatic vein (*mm*) was measured with Valsalva’s test. According to the relevant standards of imaging (Andrology branch of Chinese Medical Association, 2015; Lotti et al., 2021), grading was performed (Table 1).

**TABLE 1:** Ultrasonic testing standard.

Grade of varicocele	Palpation	Width (mm)	Reflux(s)
Normal	−	≤1.7	0
Grade 0	−	1.8–2.1	1–2
Grade I	+	2.2–2.7	2–4
Grade II	+	2.8–3.1	4–6
Grade III	+	≥3.1	≥6

### 2.10 Network Pharmacology Analysis

Under the condition of oral bioavailability (OB) ≥30%, pharmacodynamic (DL) ≥0.18, and *p* < 0.05, the active ingredients and action targets of each component of JWRJD were searched in the Symmap database (http://www.symmap.org/) and Herb database (http://herb.ac.cn/), and “varicocele” and “Male infertility” was used as keywords at the same time to retrieve the targets associated with varicocele targets in the Phenopedia database (https://phgkb.cdc.gov/PHGKB/startPagePhenoPedia.action) (Yu et al., 2008). The retrieved targets were uploaded to the Uniport database, access to target standard name. The protein species was set to *Homo sapiens* (human). The existing genes interacting with the JWRJD and varicocele were uploaded to the STRING database to obtain the molecular function (MF) pathway, cellular component (CC) pathway, and biological process (BP) pathway. Cytoscape 3.9.1 software (NIGMS, United States) was used to conduct topology analysis with the network analyzer function, and the key targets of herbal medicines were obtained by sorting according to the size of the edge-count value. At the same time, the Gene Ontology (GO) function and Kyoto Encyclopedia of Genes and Genomes (KEGG) pathway enrichment analysis were performed on the significant signaling pathways of key target genes. Finally, R language is used to construct the network of the “disease-herbs-target-pathway” through all the analyzed data.

### 2.11 Statistical Methods

GraphPad Prism 8.0 (GraphPad Software, CA, United States) was used for statistical analysis. The *K-S* test was performed on all parameters to determine the homogeneity of variance. If homogeneity of variance was normally distributed, the mean and standard deviation were used to represent it, and a *t* test of independent samples was applied. If the variance was not uniform, the quartile was used, and the chi-squared test was applied. The count and grade data were expressed as proportions and percentages, and the chi-squared test was applied. The mean values of multiple groups were compared by one-way ANOVA. Pearson correlation analysis was used to evaluate the correlation among indicators, and logistic regression analysis was used to estimate the risk of the model. Graphics was plotted by https://www.bioinformatics.com.cn, a free online platform for data analysis and visualization.

## 3 Results

### 3.1 Case Collection and Follow-Up Results

**FIGURE 1:**
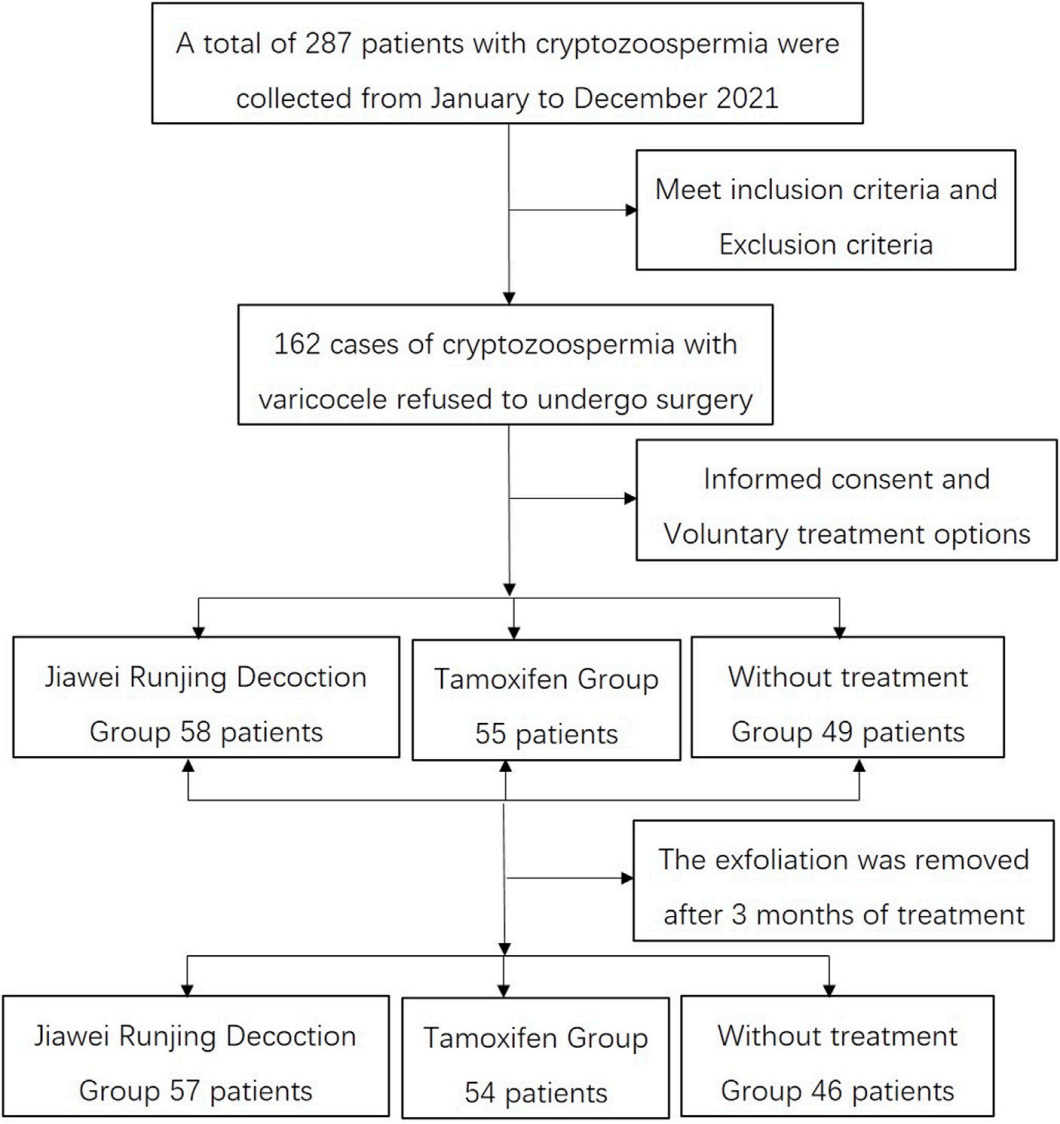
Follow-up flowchart of all the patients.

From January to December 2021, there were 287 patients with cryptozoospermia with varicoceles that were collected in two centers. In line with the inclusion criteria and exclusion criteria, only 162 patients were enrolled in the study. All patients were fully informed of treatment options and made free choices and signed informed consent forms, in which 58 patients accepted JWRJD n treatment (group A), 55 accepted tamoxifen treatment (group B), and 49 did not receive any treatment (Group C). After the 3-month follow-up, 1 patient was lost in group A, 1 in group B, and 3 in group C (Figure 1).

### 3.2 Baseline Data on the Three Groups Before the Treatment

There was no difference in the baseline data, which included age, weight, height, BMI, abstinence days, mean varicocele width, semen volume, pH, total sperm count (TSC), follicle stimulating hormone (FSH), luteinizing hormone (LH), prolactin (PRL), estradiol (E_2_), testosterone (T), neutral α-glucosidase (Neu), citric acid (CC), and zinc among the three groups before the treatment (Table 2).

**TABLE 2:** Baseline data on the three groups before the treatment.

Indicator	Group A	Group B	Group C	F	*p*
Sample size (*n*)	58	55	49	—	—
Age (*y*)	33.41 ± 4.47	34.2 ± 5.92	35.27 ± 6.10	1.504	0.225
Height (cm)	169.50 ± 2.97	169.36 ± 3.58	168.90 ± 3.18	0.489	0.614
Weight (kg)	70.78 ± 3.17	70.26 ± 2.71	71.01 ± 3.12	0.858	0.426
BMI (kg/m^2^)	24.66 ± 1.39	24.51 ± 1.09	24.90 ± 1.15	1.350	0.262
Left testicular size (ml)	13.52 ± 2.08	12.95 ± 2.84	12.76 ± 2.72	1.322	0.270
Right testicular size (ml)	14.02 ± 1.59	13.53 ± 1.99	13.43 ± 1.88	1.649	0.195
Varicocele width (mm)	2.57 ± 0.55	2.68 ± 0.61	2.74 ± 0.62	1.086	0.34
Abstinence days (*d*)	4.53 ± 1.60	4.65 ± 1.72	4.39 ± 1.59	0.320	0.727
Semen volume (ml)	3.51 ± 1.59	3.53 ± 1.86	3.46 ± 1.81	0.025	0.975
pH value	7.99 ± 0.29	8.03 ± 0.35	8.03 ± 0.33	0.267	0.766
Total sperm count (*n*)	21.36 ± 12.99	23.16 ± 17.06	22.13 ± 16.20	0.189	0.828
Follicular estrogen (mIU/ml)	14.70 ± 6.60	15.45 ± 5.94	14.21 ± 10.93	0.324	0.724
Luteinizing hormone (mIU/ml)	8.56 ± 3.21	10.22 ± 4.85	8.79 ± 6.29	1.877	0.156
Prolactin (mIU/ml)	297.19 ± 169.00	310.18 ± 156.46	244.15 ± 140.15	2.557	0.081
Estradiol (pmol/L)	107.67 ± 49.04	119.04 ± 56.13	105.36 ± 40.32	1.187	0.308
Testosterone (nmol/L)	16.18 ± 8.53	14.37 ± 7.93	12.75 ± 4.72	2.899	0.058
Neutral α-glucosidase (U/L)	36.11 ± 13.47	40.01 ± 23.36	34.42 ± 16.97	1.287	0.279
Citric acid (mmol/L)	37.53 ± 31.11	44.46 ± 32.40	40.47 ± 50.24	0.465	0.629
Zinc (mmol/L)	4.77 ± 2.91	4.97 ± 3.38	5.02 ± 5.36	0.060	0.942

### 3.3 Parameters Among the Three Groups After Treatment

**FIGURE 2:**
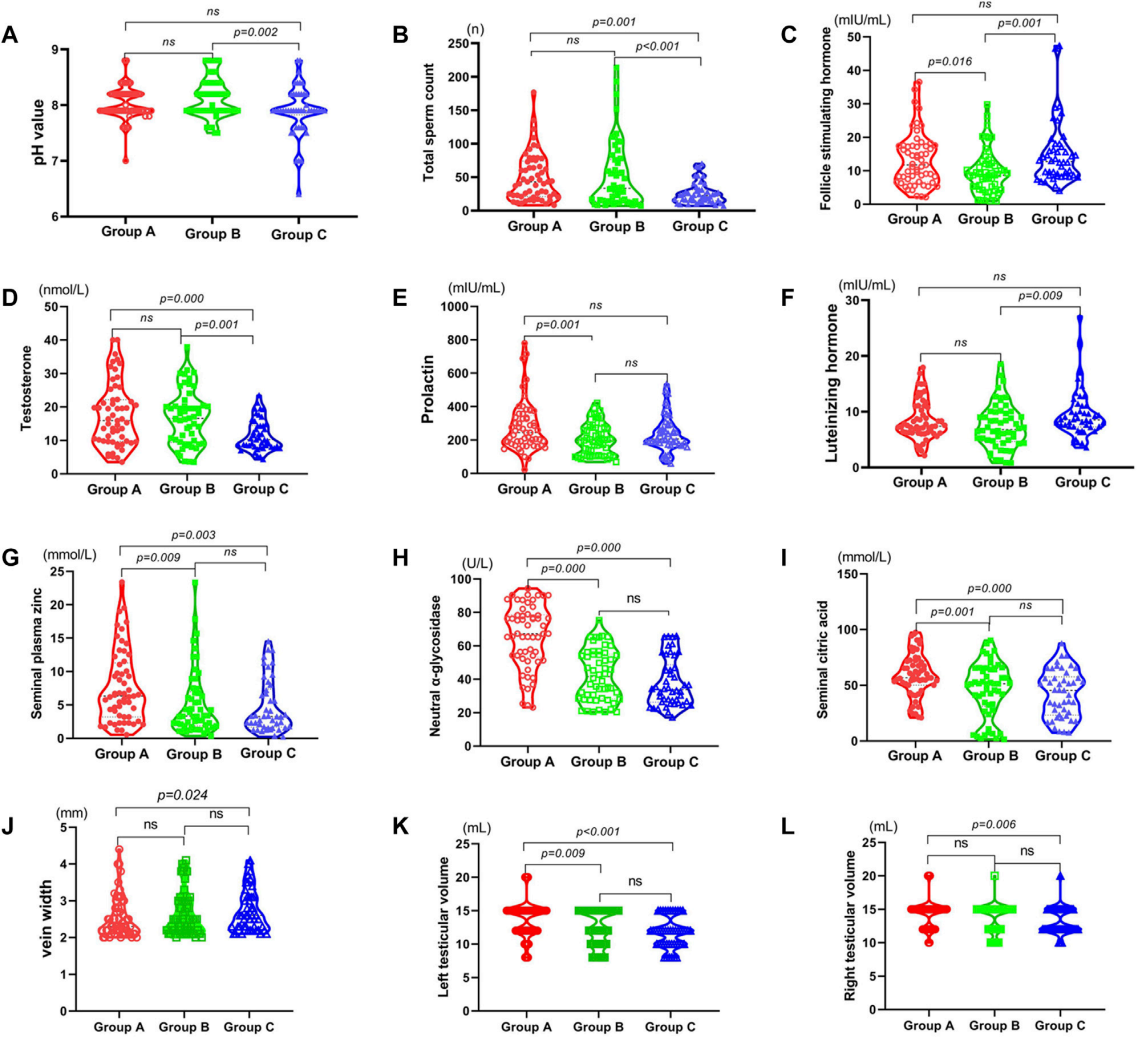
Comparison of related indicators of the three groups after treatment: red: Jiawei Runjing Decoction group (JWRJD group: group A), green: tamoxifen group (group B), and blue: no treatment group (control group: group C). **(A)** pH value. **(B)** Total sperm count. **(C)** Follicle stimulating hormone. **(D)** Testosterone. **(E)** Prolactin. **(F)** Luteinizing hormone. **(G)** Seminal plasma zinc. **(H)** Neutral α-glycosidase. **(I)** Seminal citric acid. **(J)** Vein width. **(K)** Left testicular volume. **(L)** Right testicular volume.

After treatment, the left testicle volume (L-TV), FSH, PRL, neutral α-glucosidase, citric acid, and zinc in group A were higher than those in group B (all *p* < 0.05). The total sperm count, left testicular volume, right testicular volume (R-TV), T, neutral α-glucosidase, citric acid, and zinc in group A were higher than those in group C, while the vein width (VW) was lower than that in group C (all *p* < 0.05). The total sperm count, pH, and T of group B were higher than those of group C, while LH and FSH were lower than those of group C (all *p* < 0.05). There was no difference in semen volume and E_2_ among groups A, B and C (all *p* > 0.05) (Figure 2).

### 3.4 Effect of JWRJD on Men of Different Ages

**FIGURE 3:**
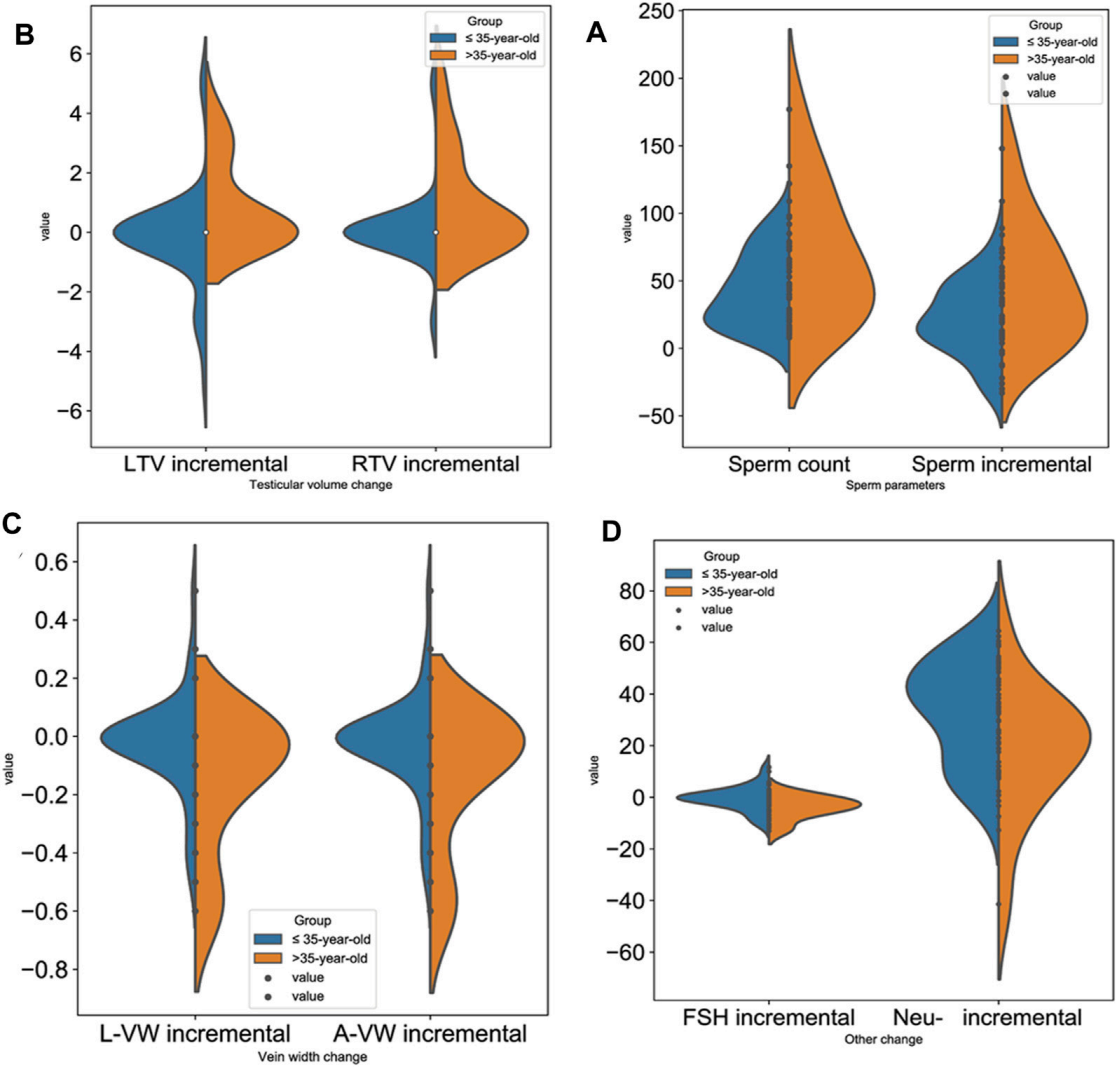
Effect of JWRJD on men of different ages. **(A)**: Sperm incremental (ΔSC): *p* < 0.05. **(B)**: Testicular volume incremental (ΔTV): *p* < 0.05. **(C)**: Vein width incremental (ΔVW): *p* < 0.05. **(D)**: Incremental FSH (ΔFSH) and neutral α-glucosidase (ΔNeu): *p* < 0.05.

Patients in group A were divided into two groups according to age to identify the effects of JWRJD: the >35-year-old group (14 patients) and the ≤35-year-old group (43 patients). The results showed that there was no difference in the patients with different ages before the treatment (*p* > 0.05) (Table 3). However, after treatment, the sperm count (SC) and the sperm incremental (ΔSC) in patients >35 years old were significantly higher than those in patients ≤35 years old (*p* < 0.05) (Figure 3). We compared the changes of the indexes in men of different ages in group A after medication. Among them, the increase in the testicular volume (ΔTV) in the >35-year-old group was greater than that in the ≤35-year-old group (Figure 3B), but there were only significant differences in the increase in the left testicular volume (ΔL-TV) (*p* < 0.05) (Figure 3B), and the increase in the width of the varicocele vein (ΔVW) was less than that in the ≤35-year-old group (*p* < 0.05) (Figure 3C), while the decrease degree of FSH (ΔFSH) was greater than that in the ≤35-year-old group (*p* < 0.05)*,* and the increase in neutral α-glucosidase (ΔNeu) was less (*p* < 0.05) (Figure 3D).

**TABLE 3:** Baseline data on different age stages in group A before the treatment.

Indicator	>35-year-old	≤35-year-old	*t*	*p*
Sample size	14	43	—	—
Height (cm)	170.57 ± 3.06	169.14 ± 2.92	1.574	0.121
Weight (kg)	69.57 ± 2.45	71.02 ± 3.19	−1.557	0.125
Varicocele width (mm)	2.75 ± 0.69	2.51 ± 0.49	1.454	0.152
Left testicular size (ml)	13.29 ± 2.30	13.67 ± 1.97	0.615	0.541
Right testicular size (ml)	13.79 ± 1.76	14.14 ± 1.54	0.722	0.473
Abstinence days (*d*)	3.93 ± 1.21	4.77 ± 1.67	−1.730	0.089
Semen volume (ml)	3.32 ± 1.57	3.58 ± 1.63	−0.518	0.607
pH value	8.06 ± 0.24	7.98 ± 0.29	0.872	0.387
Total sperm count (*n*)	21.00 ± 15.00	21.49 ± 12.46	0.121	0.904
Follicular estrogen (mIU/ml)	13.97 ± 4.12	14.88 ± 7.30	−0.442	0.660
Luteinizing hormone (mIU/ml)	8.60 ± 3.52	8.58 ± 3.17	0.023	0.982
Prolactin (mIU/ml)	295.98 ± 173.63	298.07 ± 171.51	−0.039	0.969
Estradiol (pmol/L)	110.26 ± 51.49	107.76 ± 49.02	0.164	0.870
Testosterone (nmol/L)	15.83 ± 6.80	16.07 ± 9.06	−0.090	0.929
Neutral α-glucosidase (U/L)	42.50 ± 14.92	34.32 ± 12.52	2.026	0.050
Citric acid (mmol/L)	39.66 ± 28.46	37.30 ± 32.43	0.243	0.809
Zinc (mmol/L)	5.72 ± 3.64	4.56 ± 2.58	1.315	0.194

### 3.5 Effect of JWRJD on Men With Different Varicocele Locations

**FIGURE 4:**
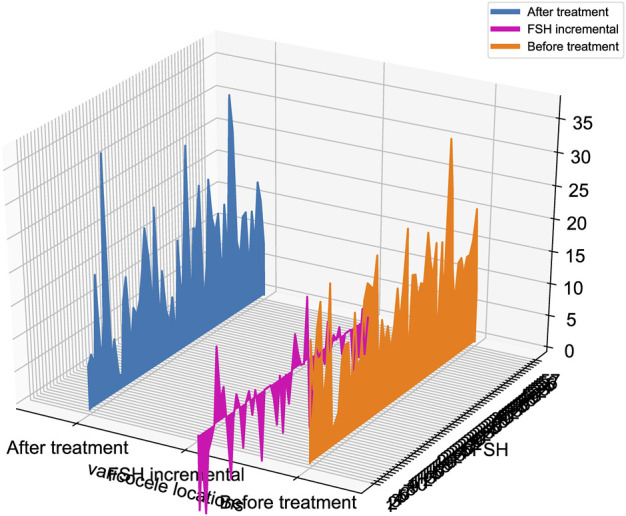
Changes of FSH (ΔFSH) before and after treatment.

Patients in group A were also divided into two groups, according to the location of varicocele to identify the effects of JWRJD. One was the unilateral varicose group, which contained 51 patients, and the other was the bilateral varicocele group, which contained 6 patients. The results showed that there was no difference in the indicators of age, weight, height, BMI, abstinence days (AD), mean varicocele width (MVW), semen volume (SV), pH value, total sperm count (TSC), follicle stimulating hormone (FSH), luteinizing hormone (LH), prolactin (PRL), estradiol (E_2_), testosterone, neutral α-glucosidase, citric acid, and zinc in patients with different varicocele locations before and after the treatment (*p* > 0.05) (Table 4). However, there were significant differences in the decrease degree of FSH (ΔFSH), and the decrease value of FSH in bilateral varicocele after the treatment was significantly higher than that in unilateral varicocele (Figure 4).

**TABLE 4:** Indicator of different locations in group A before and after the treatment.

Indicator	Before the treatment				After the treatment			
	Left side	Both sides	*t*	*p*	Left side	Both sides	*t*	*p*
Sample size (*n*)	51	6	—	—	51	6	—	—
Abstinence days (*d*)	4.61 ± 1.61	4.17 ± 1.60	0.634	0.529	4.47 ± 1.43	5.00 ± 1.79	−0.835	0.407
Semen volume (ml)	3.49 ± 1.60	3.70 ± 1.79	−0.294	0.770	3.29 ± 1.62	2.30 ± 0.71	1.472	0.147
pH value	8.01 ± 0.28	7.92 ± 0.29	0.764	0.448	8.04 ± 0.32	7.90 ± 0.19	1.131	0.263
Total sperm count (*n*)	20.51 ± 12.45	28.67 ± 16.38	−1.470	0.147	48.35 ± 34.28	60.33 ± 45.74	−0.782	0.437
Follicular estrogen (mIU/ml)	14.59 ± 6.77	15.24 ± 6.01	−0.225	0.823	13.61 ± 8.03	9.78 ± 5.32	1.133	0.262
Luteinizing hormone (mIU/ml)	8.36 ± 3.18	10.50 ± 3.23	−1.555	0.126	8.66 ± 3.65	7.38 ± 2.65	0.832	0.409
Prolactin (mIU/ml)	297.92 ± 176.67	294.43 ± 115.54	0.047	0.963	279.94 ± 155.43	282.88 ± 96.77	−0.045	0.964
Estradiol (pmol/L)	104.11 ± 45.65	144.58 ± 67.03	−1.954	0.056	104.06 ± 53.57	136.57 ± 82.85	−1.325	0.191
Testosterone (nmol/L)	15.89 ± 8.58	17.04 ± 8.53	−0.310	0.758	17.30 ± 9.43	19.94 ± 10.71	−0.641	0.524
Neutral α-glucosidase (U/L)	35.35 ± 13.24	44.65 ± 13.83	−1.621	0.111	66.75 ± 17.82	57.96 ± 27.32	1.078	0.286
Citric acid (mmol/L)	38.71 ± 32.88	30.84 ± 8.41	0.580	0.565	60.06 ± 19.53	52.82 ± 17.65	0.866	0.390
Zinc (mmol/L)	4.60 ± 2.73	6.91 ± 3.62	−1.900	0.063	7.98 ± 5.55	5.37 ± 4.01	1.113	0.271

### 3.6 Effect of JWRJD on Men With Different Varicocele Grades

Patients in group A were also divided into four groups, according to the grade of varicocele to identify the effects of JWRJD. The grade 0 group contained 15 patients, the grade I group contained 27 patients, the grade II group contained 4 patients, and the grade III group contained 11 patients.

**FIGURE 5:**
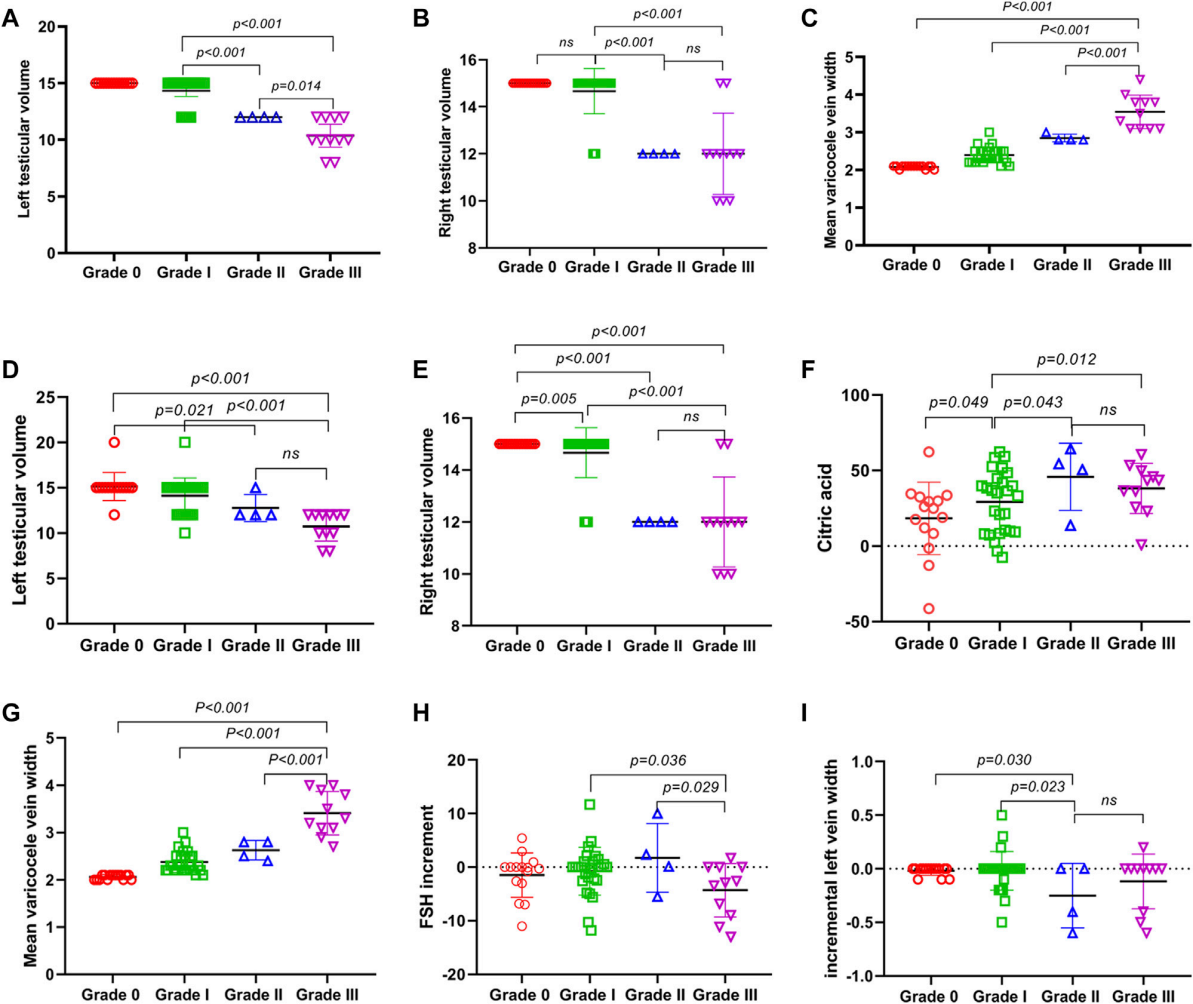
Effect of JWRJD on men with different varicocele grades. **(A)** Left testicular volume before treatment. **(B)** Right testicular volume before treatment. **(C)** Mean varicocele vein width before treatment. **(D)** Left testicular volume after treatment. **(E)** Right testicular volume after treatment. **(F)** citric acid after treatment. **(G)** mean varicocele vein width after treatment. **(H)** incremental FSH after treatment. **(I)** incremental left vein width after treatment.

There was no difference in age, height, weight, BMI, abstinence days, semen volume, total sperm count (*TSC*), hormone (including follicle stimulating hormone, luteinizing hormone, prolactin, estradiol, and testosterone), and seminal plasma biochemical components (including neutral α-glucosidase, citric acid, and zinc) of the patients with different varicocele locations before treatment (*p* > 0.05) (Table 5). However, there were significant differences in the testicular volume (TV) (Figures 5A,B) and varicocele vein width (VW) (Figure 5C).

**TABLE 5:** Indicator of different grades in group A before the treatment.

Indicator	Grade 0	Grade I	Grade II	Grade III	F	*p*
Sample size (*n*)	15	19	10	13	—	—
Age (*y*)	33.73 ± 5.61	32.95 ± 4.36	34.00 ± 2.94	33.92 ± 4.03	0.188	0.904
Height (cm)	168.87 ± 3.36	170.00 ± 2.43	169.40 ± 2.41	169.51 ± 3.82	0.391	0.760
Weight (kg)	72.10 ± 2.92	70.45 ± 3.16	69.25 ± 3.11	70.42 ± 2.72	1.957	0.132
BMI (kg/m^2^)	25.30 ± 1.26	24.40 ± 1.49	24.15 ± 1.29	24.52 ± 1.22	1.919	0.138
Abstinence days (*d*)	4.33 ± 1.45	4.42 ± 1.61	5.40 ± 1.78	4.38 ± 1.61	1.121	0.349
Semen volume (ml)	4.07 ± 1.85	3.23 ± 1.44	4.09 ± 1.38	2.86 ± 1.51	2.046	0.119
pH value	7.99 ± 0.30	8.02 ± 0.26	7.97 ± 0.37	8.00 ± 0.23	0.072	0.975
Total sperm count (*n*)	19.80 ± 10.60	18.74 ± 12.65	22.70 ± 14.45	26.00 ± 14.86	0.915	0.440
Follicular estrogen (mIU/ml)	14.75 ± 6.29	12.81 ± 7.38	14.25 ± 7.64	17.58 ± 4.44	1.371	0.262
Luteinizing hormone (mIU/ml)	8.14 ± 3.16	8.59 ± 2.39	7.79 ± 4.70	9.71 ± 3.11	0.817	0.490
Prolactin (mIU/ml)	247.09 ± 185.78	331.47 ± 191.44	287.16 ± 126.06	314.21 ± 151.89	0.723	0.538
Estradiol (pmol/L)	100.44 ± 34.11	104.65 ± 53.16	98.77 ± 34.17	130.36 ± 64.15	1.170	0.330
Testosterone (nmol/L)	17.43 ± 7.57	14.93 ± 9.93	14.18 ± 8.69	17.36 ± 7.55	0.493	0.689
Neutral α-glucosidase (U/L)	34.39 ± 12.70	36.57 ± 13.78	45.30 ± 16.31	31.32 ± 8.81	2.334	0.084
Citric acid (mmol/L)	47.12 ± 25.39	41.52 ± 38.15	43.47 ± 33.48	17.60 ± 13.72	2.671	0.057
Zinc (mmol/L)	5.98 ± 2.96	4.43 ± 2.15	5.35 ± 3.39	3.73 ± 3.12	1.707	0.177

After treatment, the testicular volume (TV) (Figures 5D,E) in grade III was lower than grade I and grade 0, but without difference with grade II. The mean varicocele vein width (Figure 5G) in grade III was greater than grade II and grade I, but the incremental FSH (ΔFSH) (Figure 5H) was lower. In grade II, the incremental left vein width (ΔL-VW) (Figure 5I) were less than grade I and grade 0, but the citric acid (CC) were greater (Figure 5F), and without difference with grade III.

### 3.7 Factors Affecting the Therapeutic Effect of JWRJD

**FIGURE 6:**
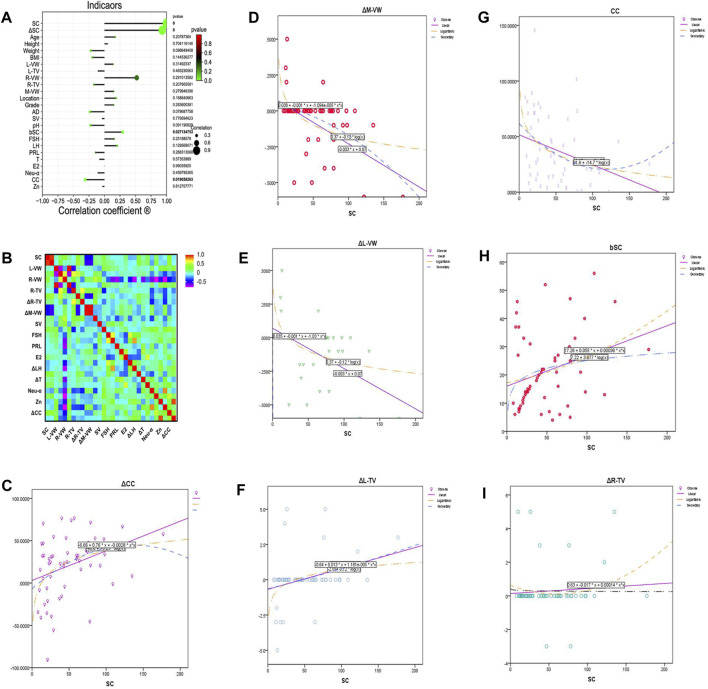
Factors affecting the therapeutic effect of JWRJD. **(A)** Correlation between sperm count after treatment and other indicators. **(B)** Relationship between total sperm count increment and other indicators. **(C)** Relationship between SC and ΔCC. **(D)** Relationship between SC and ΔM-VW. **(E)** Relationship between SC and ΔL-VW. **(F)** Relationship between SC and ΔL-TV. **(G)** Relationship between SC and CC. **(H)** Relationship between SC and bSC. **(I)** Relationship between SC and ΔR-TV.

Pearson correlation analysis was performed for group A to identify the related factors affecting the therapeutic effect of JWRJD. The sperm count after treatment with JWRJD was related to the sperm count before treatment (bSC) (*r =* 0.293; *p =* 0.027; 95% CI: 0.0348 to 0.541), CC (*r* = −0.310; *p* = 0.019; 95% CI:−0.528 to −0.0535), ΔL-TV (*r =*0.317; *p* = 0.016; 95% CI: 0.062 to 0.534), ΔL-VW (*r =*−0.474; *p* < 0.001; 95% CI: −0.660 to −0.244), ΔM-VW (I = −0.482; *p* < 0.001; 95% CI:−0.660 to −0.254), and ΔCC (*r* =0.344; *p* = 0.009; 95% CI: 0.0915 to 0.555) (Figure 6A). ΔSC was related to CC (*r* = −0.269; *p* = 0.043; 95% CI: −0.495 to −0.010), ΔL-TV (*r* = 0.421; *p* = 0.001; 95% CI: 0.181–0.615), ΔL-VW (*r* = −0.539; *p* < 0.001; 95% CI:−0.701 to −0.323), ΔM-VW (*r* = −0.546; *p* < 0.001; 95% CI:−0.706 to −0.333), and ΔCC (*r* = −0.090; *p* = 0.007; 95% CI: 0.0997–0.560) (Figure 6B).

Linear regression equation analysis, logarithmic regression equation analysis, and quadratic regression equation analysis were performed to analyze the predictive value of seven related indicators (Table 6); it was found that ΔCC (Figure 6C), ΔM-VW (Figure 6D), ΔL-VW (Figure 6E), ΔL-TV (Figure 6F), CC (Figure 6G), and bSC (Figure 6H) were significant indexes to predict the therapeutic effect except ΔR-TV (Figure 6I).

**TABLE 6:** Regression analysis of related indicators.

Dependent index	Equation	R^2^	F	df1	df2	Sig	Constant	b1	b2
ΔCC	Linear	0.118	7.375	1	55	0.009	3.059	0.352	
ΔM-VW	Linear	0.233	16.667	1	55	0	0.075	−0.003	
ΔR-TV	Linear	0.006	0.356	1	55	0.553	0.14	0.003	
ΔL-TV	Linear	0.101	6.161	1	55	0.016	−0.683	0.015	
CC	Linear	0.096	5.835	1	55	0.019	51.556	−0.273	
bSC	Linear	0.086	5.154	1	55	0.027	16.033	0.108	
ΔCC	Logarithmic	0.142	9.104	1	55	0.004	−46.094	18.253	
ΔM-VW	Logarithmic	0.209	14.549	1	55	0	0.372	−0.117	
ΔR-TV	Logarithmic	0	0.011	1	55	0.919	0.393	−0.026	
ΔL-TV	Logarithmic	0.108	6.655	1	55	0.013	−2.6	0.727	
CC	Logarithmic	0.124	7.795	1	55	0.007	91.668	−14.705	
bSC	Logarithmic	0.05	2.876	1	55	0.096	7.22	3.877	
ΔCC	Secondary	0.139	4.370	2	54	0.017	−6.664	0.762	−0.003
ΔM-VW	Secondary	0.243	8.688	2	54	0.001	0.038	−0.001	−1.09E-05
ΔR-TV	Secondary	0.043	1.2	2	54	0.309	0.634	−0.018	0
ΔL-TV	Secondary	0.101	3.03	2	54	0.057	-0.643	0.013	1.18E-05
CC	Secondary	0.126	3.891	2	54	0.026	61.562	−0.695	0.003
bSC	Secondary	0.088	2.615	2	54	0.082	17.262	0.056	0

### 3.8 Network Pharmacology Analysis

**FIGURE 7:**
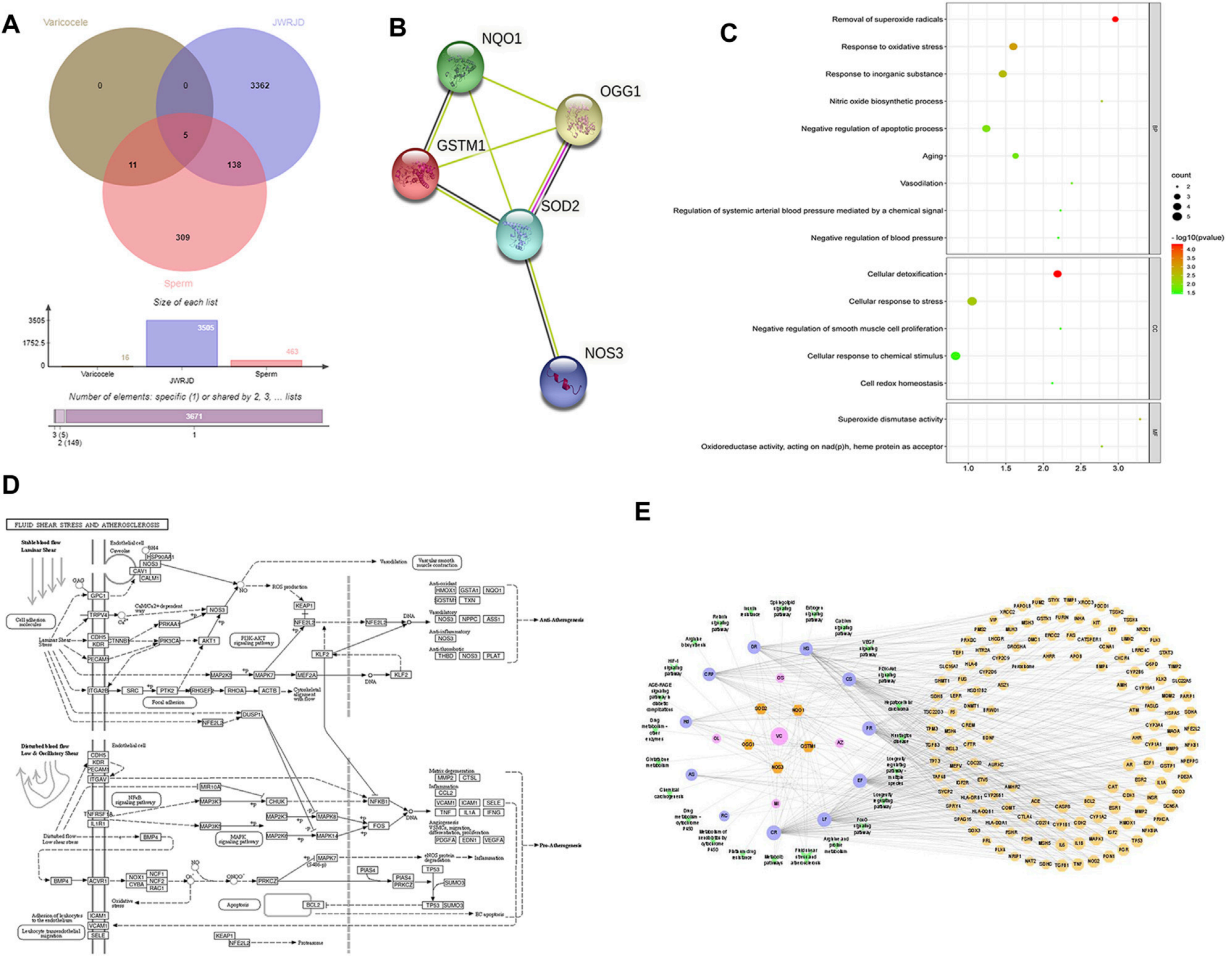
Network pharmacology analysis: **(A)**: Venn diagram of common target genes. **(B)**: PPI network map of common target genes. **(C)**: GO function bubble diagram. **(D)**: KEGG pathway chart. **(E)**: Drug-disease-target-pathway network map.

A total of 397 active compounds and 3,505 action targets of JWRJD were obtained; combined with 461 spermatogenesis-related gene targets [includes four diseases: asthenozoospermia (OS), azoospermia (AZ), male infertility (MI), oligospermia (OL)] and 16 varicocele (VC)-related gene targets, there were five common targets between the three which contained GSTM1, NQO1, NOS3, OGG1, and SOD2 (Figure 7A). The common targets were uploaded to the STRING database, the PPI network diagram of the common target was obtained, and seven edges were obtained altogether (PPI enrichment *p*-value: 7.11e-08) (Figure 7B). A total of 16 items were obtained from GO function analysis, including nine items from biological process analysis, five items from cell component analysis, and two items from molecular function analysis. According to the order of *p* value from small to large, “among all the items: clear superoxide radical, cellular detoxification, reaction to oxidative stress, superoxide dismutase activity, and reaction to inorganic substances” ranked in the top five (Figure 7C). In total, 24 signal pathways were obtained by KEGG pathway enrichment analysis and were sorted from small to large according to the *p* value. “Fluid shear stress and atherosclerosis, AGE-RAGE signaling pathway in diabetic complications, FoxO signaling pathway, platinum drug resistance, and HIF-1 signaling pathway” ranked in the top five. Fluid shear stress and atherosclerosis, which affect the internal environment, are the most significant (Figure 7D). Based on these results, the drug-disease-target-pathway network map was successfully constructed (Figure 7E).

## 4 Discussion

Cryptozoospermia with varicocele is a multifactor disease combining low spermatogenic function and varicocele. Whether it can be recovered after surgery is still controversial (Jarow, 2001; Lira Neto et al., 2021; Ming Yan et al., 2021). Therefore, many patients prefer to give up surgery and seek medical treatment or other ways to assist pregnancy (Zhao et al., 2021). Among our 287 patients, 162 (56.4%) gave up surgical treatment. There were two main factors in our analysis. First, the high cost of surgery may be the main factor affecting the selection of patients (Balasubramanian et al., 2019; Wickham et al., 2021). After surgery, some patients’ sperm still cannot return to the normal state or become pregnant naturally, so they still need to seek other alternatives. Second, the traumatic nature of surgery is one of the main factors. Studies have shown that men would have significantly higher fear than normal when they were on the condition of disease (Jayadevan et al., 2020; Minhas et al., 2021). Infertile men with cryptozoospermia would especially conceal; when they faced unsuccessful facts, they would have much more depression and anxiety, and the fear of surgical trauma would increase (Xu et al., 2019; Yang et al., 2017). Therefore, most people will seek conservative or non-invasive means. The development of JWRJD meets the needs of these patients, which can help patients improve the sperm count and pregnancy rate as much as possible without surgery.

Tamoxifen (Cannarella et al., 2019; Dimakopoulou et al., 2021), letrozole (Kooshesh et al., 2020), and L-carnitine (Zhang et al., 2020) are commonly used in the treatment of oligozoospermia. There have also been reports and studies on the treatment of cryptozoospermia (Tadros and Sabanegh, 2017), but the effect has not been recognized (Garg and Kumar, 2015; Semet et al., 2017). Chinese herbal remedies have been used to treat infertility in China for thousands of years, although their mechanism has not been proven by evidence-based medical theory (Huang and Chen, 2008; Zhu et al., 2018; Jiang et al., 2021). According to TCM, varicocele belongs to *Qi-stagnation and Blood-stasis* syndrome. Some patients are complicated with dampness and heat injection (Dun et al., 2015; Yu et al., 2019; Jiang et al., 2021). At the same time, TCM also believes that low spermatogenesis is rooted in the *Shen* (kidney). The insufficiency of *Shen-Yang* and deficiency of *Shen-Yin* will cause poor circulation of *Qi* and *Blood* and metabolic disorders, which would result in low spermatogenesis (Tempest et al., 2008; Jiang et al., 2017; Sylvia Yan et al., 2021). In JWRJD, CS, EF, and HS are king drugs that are used to nourish *Shen-Yang* and promote spermatozoa production (Anand Ganapathy et al., 2021). PR, DR, LF, and CRP are minister medicines used for nourishing *Yin-Qi* and spleen and nourishing sperm development (Chen et al., 2021). RC, CR, and AS are adjuvant drugs that are used to tonify Qi and Essence, promote Qi and blood circulation, remove blood stasis, and enhance sperm maturation (Sandhyakumary et al., 2002). Hd and ES are used to break blood and remove blood-stasis, activate collaterals and channels, and promote sperm excretion (Ma et al., 2019). In our observation, the results showed that the total sperm count could significantly increase after treatment with JWRJD and tamoxifen. The sperm count and testosterone level of these two groups were significantly higher than those of the patients without any treatment. Therefore, we suggest that both Chinese herbal medicine and tamoxifen would effectively improve the sperm count in cryptozoospermia patients.

However, the total sperm count was not different between the JWRJD group and the tamoxifen group. We compared various indicators affecting the treatment effect and found that after tamoxifen treatment, FSH significantly decreased and testosterone increased, revealing that the way tamoxifen increased the total sperm count might be through regulating the sex hormone mechanism. After treatment with JWRJD, although testosterone showed no signs of increasing, FSH decreased significantly, and through the negative feedback, which might stimulate testosterone secretion. We confirmed that the method of improving the sperm total by JWRJD might be related to the sex hormone mechanism. This is similar to the tamoxifen which was considered to be the master of boosting sperm counts (Balasubramanian et al., 2019; Cannarella et al., 2019; Sadeghi et al., 2019). When we want to treat men with cryptozoospermia of varicocele with drugs, JWRJD may increase the testosterone concentration, which can be a priority choice.

To further understand how it works, we compared each indicator of seminal plasma biochemistry in the three groups in depth. We found that the seminal neutral α-glucosidase, citric acid, and zinc played the important role, revealing that the increase in the sperm total might be related to the change in seminal plasma components, especially the increase in seminal plasma neutral α-glucosidase, citric acid, and zinc. Zinc is the second most essential trace element in the human body and is involved in the whole process from spermatogenesis to childbirth (Vickram et al., 2021). Zinc plays an important role in male fertility, especially in seminal plasma, and the concentration of seminal plasma zinc is closely related to the sperm concentration (Osadchuk et al., 2021). Studies have shown that zinc deficiency may lead to sperm decline through oxidative stress and apoptosis (Beigi Harchegani et al., 2020). Animal experiments have also confirmed that zinc can activate MAPK and promote sperm cell apoptosis by exacerbating oxidative damage (Chen et al., 2020). Therefore, combined with our observations, we highly suspect that JWRJD increases spermatogenesis by increasing the concentration of seminal plasma zinc.

Studies have suggested that there is a difference in spermatogenesis between unilateral varicocele and bilateral varicocele after treatment (Niu et al., 2018; Ou et al., 2019). One prospective randomized controlled study found that bilateral varicocele surgery was more effective than unilateral surgery (Sun et al., 2018). In our study, we also compared the therapeutic effects of unilateral varicocele and bilateral varicocele and found that there were no differences in semen parameters, reproductive hormones, or seminal plasma biochemical indexes of the two different types of patients when they were treated with JWRJD. In our opinion, the main reason for this may be the mechanism of TCM treatment. TCM treatment emphasized the overall effect and changed the *Yin-Yang* and *Qi-Blood* (Fu et al., 2021). The king medicine in JWRJD regulates the *Shen-Yin* and *Shen-Yang*, and the minister medicine improves *Qi* and *Blood*, and the adjuvant medicine improves the function of the liver and spleen to maximize the spermatogenic function; thus, JWRJD can produce a different principle from tamoxifen.

Some studies have suggested that different grades of varicocele have different effects of surgical treatment. The surgical effect of grade 1 patients was inferior to that of grade 2–3 patients (Asafu-Adjei et al., 2020). Some studies also suggest that surgery is more statistically significant for patients with higher grades of varicocele (Vahidi et al., 2018), and high-grade left varicocele surgery is more effective (Grasso et al., 2014). In our study, we also found unexpected and similar results. We found that although the location of varicocele did not affect the therapeutic effect of JWRJD, the grades and width of varicocele were closely related to the therapeutic effect of JWRJD. After treatment, the sperm count in patients with grade III varicoceles was significantly higher than that in patients with grade 0 and II varicoceles in the JWRJD group. Previous studies have confirmed that the left grade III varicocele could affect epididymis function, which could be improved after treatment (Lehtihet et al., 2014). A study of 623 Russian men found a positive correlation between the sperm count and zinc concentration in seminal plasma (Osadchuk et al., 2021). We also found that the seminal plasma composition in patients with grade III varicoceles was significantly higher than that in patients with grade II varicoceles in the JWRJD group. This revealed that JWRJD had a more ideal effect on higher grade varicocele patients, and its main effect was to improve the concentration of the seminal plasma composition, so we believed this also confirmed again that JWRJD could improve the sperm count by regulating the seminal plasma composition.

Age is the main factor affecting spermatogenesis, which has been confirmed in many studies (Cioppi et al., 2019; Frungieri et al., 2021; Frungieri.et al., 2021). Age is also an important factor for aggravating varicoceles (Kimura et al., 2017; Bolat et al., 2019; Zampieri et al., 2021). The effect of spermatogenesis drug intervention in men of different ages has not been recognized (Palmisano et al., 2019; Kohn et al., 2020). Animal experiments also confirmed that zinc concentrations varied in different seasons and ages (Kandiel and El Khawagah, 2018). In our study, we analyzed the effect of drug therapy on cryptozoospermia of varicocele in different age groups. We found that JWRJD had different effects on males of different ages, and the sperm count in patients >35 years old was significantly higher than that in those ≤35 years old. This also inspired us to look for the cause of this phenomenon. Previous animal studies have found that the Sertoli cell function is different in rats of different ages, which could lead to large differences in FSH and spermatogenesis (Castellon et al., 1989). FSH concentration is closely related to fertility in young men (Lindgren et al., 2012). A study involving 2,448 men found a strong correlation between the FSH concentration and sperm quality in men of different ages (Grunewald et al., 2013). We compared the reproductive hormones of men of different ages in the JWRJD treatment group and found that FSH in patients >35 years old was significantly lower than that in those ≤ 35 years old. This meant that JWRJD could significantly reduce FSH levels in older men and promote spermatogenesis.

Network pharmacology has given us good hints and verification. Through network pharmacology analysis, we found that JWRJD can regulate spermatogenesis in patients with varicocele spermatogenesis by improving the fluid shear stress and atherosclerosis pathway. The changes in blood flow in patients with varicocele reflect the changes in the testicular microenvironment. Therefore, JWRJD may improve the sperm quality by improving the testicular microenvironment.

Considering age, grades of varicoceles, reproductive hormones, and seminal plasma composition, we found that JWRJD could regulate reproductive hormones and seminal plasma composition in men of different ages and grades to improve the sperm count, which is worthy of promotion and application.

## 5 Conclusion

JWRJD may promote spermatogenesis in cryptozoospermia patients with varicocele, which may be closely related to improving the testicular microenvironment, especially for >35-year-old and grade III varicocele patients.

## Data Availability

The original contributions presented in the study are included in the article/Supplementary Material; further inquiries can be directed to the corresponding authors.

## Glossary

SERMs: selective estrogen receptor modulators
LH: luteinizing hormone
TCM: traditional Chinese medicine
RJD: Runjing Decoction
JWRJD: Jiawei Runjing Decoction
WHO: World Health Organization
AZF: azoospermia factor
FSH: follicle stimulating hormone
ΔFSH: increment of the follicle stimulating hormone
PRL: prolactin
E2: estradiol
T: testosterone
OB: oral bioavailability
DL: pharmacodynamic
MF: molecular function
CC: cellular component: citric acid
BP: biological process
GO: Gene Ontology
KEGG: Kyoto Encyclopedia of Genes and Genomes
BMI: body mass index
AD: abstinence days
MVW: mean varicocele width
L-VW: left varicocele width
R-VW: right varicocele width
ΔM-VW: increment of the mean varicocele width
ΔL-VW: increment of the left varicocele width
ΔR-VW: increment of the right varicocele width
SV: semen volume
TSC: total sperm count
SC: sperm count
bSC: sperm count before the treatment
ΔSC: increment of the sperm count
Neu: neutral α-glucosidase
ΔNeu: increase in neutral α-glucosidase
CC: cellular component: citric acid
ΔCC: increment of citric acid
Zn: zinc
TV: testicular volume
L-TV: left testicular volume
ΔL-TV: increment of the left testicular volume
R-TV: right testicular volume
ΔR-TV: increment of the right testicular volume
GSTM1: glutathione S-transferase M1
NQO1: quinone oxidoreductase 1
NOS3: nitric oxide synthase 3
OGG1: 8-oxoguanine DNA glycosylase
SOD2: superoxide dismutase 2
CS: *Cuscuta chinensis* Lam.
DR: *Dioscorea polystachya* Turcz.
PR: *Polygonatum sibiricum* Redouté
EF: *Epimedium brevicornu* Maxim.
LF: *Lycium barbarum* L.
AS: *Eleutherococcus senticosus* (Rupr. & Maxim.) Maxim.
RC: *Rhodiola crenulata* (Hook.f. & Thomson) H.Ohba
CR: *Cyathula officinalis* K.C.Kuan
CRP: *Citrus aurantium* L.
Hd: Hirudo
HS: *Homo sapiens*
ES: Eupolyphaga seu Steleophaga
OS: asthenozoospermia
AZ: azoospermia
MI: male infertility
OL: oligospermia
VC: varicocele
